# Retrospective exploration of risk factors for L5 radiculopathy following lumbar floating fusion surgery

**DOI:** 10.1186/s13018-015-0307-4

**Published:** 2015-10-17

**Authors:** Sumihisa Orita, Masatsune Yamagata, Yoshikazu Ikeda, Fumitake Nakajima, Yasuchika Aoki, Junichi Nakamura, Kazuhisa Takahashi, Takane Suzuki, Seiji Ohtori

**Affiliations:** Department of Orthopaedic Surgery, Graduate School of Medicine, Chiba University, Chiba, Japan; Department of Orthopaedic Surgery, Chiba Rosai Hospital, 1-8-1 Inohana, Chuo-ku, Chiba, 260-8670 Japan; Department of Orthopaedic Surgery, East Chiba Medical Center, Chiba, Japan; Department of Bioenvironmental Medicine, Graduate School of Medicine, Chiba University, Chiba, Japan

**Keywords:** Floating fusion surgery, Adjacent segment disorder (ASD), Clinical outcome, L5 spinal nerve disorder, Radiculopathy

## Abstract

**Background:**

Lumbar floating fusion occasionally causes postoperative adjacent segment disorder (ASD) at lumbosacral level, causing L5 spinal nerve disorder by L5-S1 foraminal stenosis. The disorder is considered to be one of the major outcomes of L5-S1 ASD, which has not been evaluated yet. The present study aimed to evaluate the incidence and risk factors of postoperative L5 spinal nerve disorder after lumbar interbody fusion extending to the L5 vertebra.

**Methods:**

We evaluated 125 patients with a diagnosis of spondylolisthesis who underwent floating fusion surgery with transforaminal lumbar interbody fusion with average postoperative period of 25.2 months. The patients were regarded as symptomatic with postoperative L5 spinal nerve disorder such as radicular pain/numbness in the lower limbs and/or motor dysfunction. We estimated and compared the wedging angle (frontal view) and height (lateral view) of the lumbosacral junction in pre- and postoperative plain X-ray images and the foraminal ratio (ratio of the narrower foraminal diameter to the wider diameter in the craniocaudal direction) in the preoperative magnetic resonance image. Risk factors for the incidence of L5 spinal nerve disorder were explored using multivariate logistic regression.

**Results:**

Eight of the 125 patients (6.4 %) were categorized as symptomatic, an average of 13.3 months after surgery. The wedging angle was significantly higher, and the foraminal ratio was significantly decreased in the symptomatic group (both *P* < 0.05) compared to the asymptomatic group. Multivariate logistic regression analysis of possible risk factors revealed that the wedging angle, foraminal ratio, and multileveled fusion were statistically significant.

**Conclusions:**

Higher wedging angle and lower foraminal ratio in the lumbosacral junction were significantly predictive for the incidence of L5 nerve root disorder as well as multiple-leveled fusion. These findings indicate that lumbosacral fixation should be considered for patients with these risk factors even if they have few symptoms from the L5-S1 junction.

## Background

Recent developments in spinal instrumentation have enabled more stable and multilevel fusion in degenerative spondylolisthesis patients. Some patients with no symptom from L5-S1 junction undergo lumbar floating fusion surgery terminating at the L5 level. Herein, the indication for L5-S1 arthrodesis in patients with an asymptomatic L5-S1 junction is sometimes controversial [1–4].

One study strongly suggests routine L5-S1 fusion to decrease pain and preserve lumbar function [5], while others maintain that asymptomatic patients need no fusion [6, 7]. One reason for the controversy is the presence of adjacent segment disease (ASD), which mainly occurs at the adjacent intervertebral disc after fusion surgery and decreases adjacent intervertebral disc height. The overall occurrence rate of ASD is reported to be almost as much as 50 % when caudal and cranial ASD are considered together [2]. Herein, the L5-S1 junction is an isolated intervertebral disc space functioning as the most inferior inflection point in spinal alignment; as such, it is overexposed to a large amount of load, leading to L5-S1 intervertebral disc degeneration, which is impossible to be anticipated before surgery [8]. Thus, some previous studies have suggested a conclusion that patients with sagittal imbalance and lumbar hypolordosis should undergo L5-S1 fusion even with minimal L5-S1 disc degeneration [5]. In addition to disc degeneration, ASD includes additional pathologies such as instability, listhesis, facet joint hypertrophy, herniated nucleus pulposus, and stenosis. In particular, a degenerated and herniated L5-S1 disc can lead to L5-S1 foraminal stenosis followed by consequent impingement of the L5 spinal nerve [9, 10]. The symptom sometimes gives postoperative patients severe distress requiring revision surgery; however, its clinical incidence is unclear as ASD itself is sometimes asymptomatic.

In this retrospective study, we explored the prevalence and risk factors for L5 spinal nerve disorder as the primary outcome after floating fusion surgery.

## Methods

### Patient selection and surgical indication

Following institutional review board approval, 125 adult patients who underwent primary posterior lumbar decompression and instrumented transforaminal lumbar interbody fusion (TLIF) stopping inferiorly at L5 were included in the study; surgeries were conducted between January 2005 and December 2008. Informed consent to participate in the study should be obtained from participants. Patients were diagnosed with spondylolisthesis of >5 % in the neutral position at L4 or above with instability of one translation ≥5 mm and posterior instability ≥ 5° in flexion. The patients were diagnosed from images, including those obtained from magnetic resonance (MR) imaging, and symptoms such as intermittent neural claudication and intractable lower back pain. Patients with L5 nerve root disorder from apparent L5-S1 foraminal stenosis in MR sagittal T1-weighted images (WI) [11] were excluded, as they clinically need lumbosacral foraminotomy, such as L5-S1 TLIF surgery. The indications for fusion surgery were spondylolisthesis with the translational change described above, progression of deformity, and intractable leg pain. Patients with systemic complications that can affect the outcome, such as DISH, diabetes mellitus (HbA1c ≥ 6.0 %), transitional vertebrae, and kyphoscoliosis, were excluded.

### Evaluation

The primary observations in the present study included the incidence of postoperative L5 radiculopathy coincident to the L5 dermatome, and/or motor dysfunction of the anterior tibialis and/or extensor hallucis longus muscle that was not present preoperatively. The symptom was confirmed by a physical finding of L5 dermatomal pain or numbness after the surgery, which was also improved by L5 nerve root infiltration. In addition, it was confirmed using MR imaging showing a decompressed L4-L5 canals in the axial T2 WI and L5-S1 foraminal stenosis in the sagittal T1 WI. The radiological evaluation was performed by three individual spine surgeons.

Patients who underwent surgery were divided into two groups according to the incidence of postoperative L5 radiculopathy during the follow-up: a symptomatic group (Sym), in which the patients showed L5 radiculopathy, and an asymptomatic group (Asym) with no symptoms of L5 radiculopathy.

### Surgical technique

Patients underwent TLIF surgery with bilateral decompression using a hemi-open approach followed by interbody fusion using the Legacy Spinal System (Medtronic Sofamor Danek, Memphis, TN) for pedicle screws and OIC PEEK cage (Stryker, Kalamazoo, MI) filled with local bone graft for the interbody cage by four spine surgeons. Pedicle screws in the opposite side were inserted using the Wiltse approach.

### Radiographic parameters

**Fig. 1:**
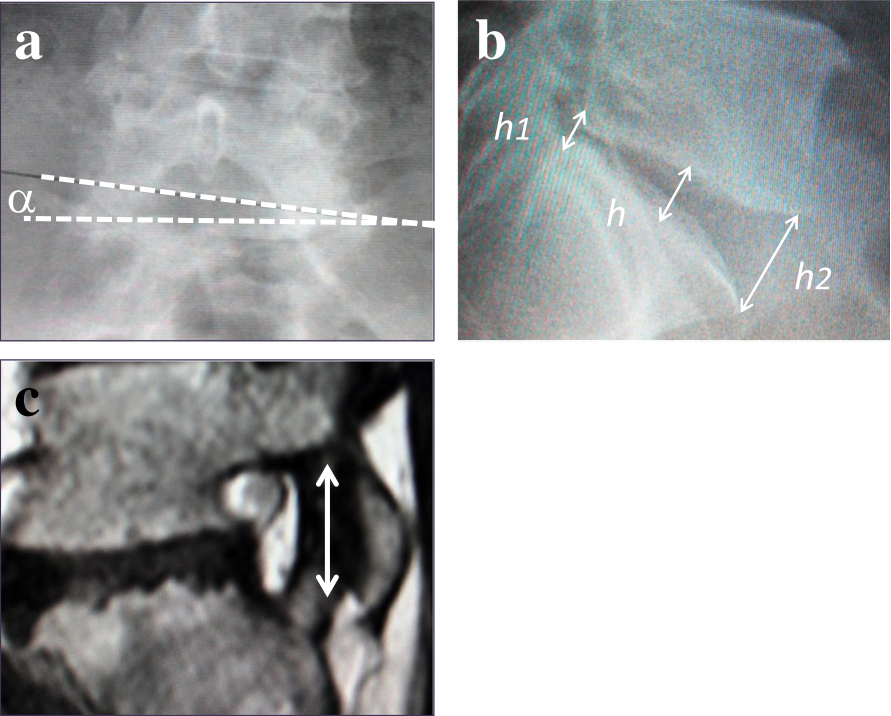
Radiographic parameters. **a** L5-S1 wedging angle in a frontal standing plain X-ray. **b** Disc height of L5-S1 intervertebral disc: *h* = (*h1* + *h2*) × 1/2. **c** Foraminal ratio: bilateral L5-S1 foraminal ratio of the narrower to the wider foraminal craniocaudal diameter

The pre- and postoperative wedging angle in the frontal view, lumbosacral height in the sagittal view in the standing lumbar X-ray image, and the bilateral foraminal ratio in the sagittal T1 WI in MR imaging were measured as radiographic parameters (Fig. 1). Instability at the final follow-up was also evaluated using plain X-ray.

## Statistical analysis

The parameters were statistically evaluated using Mann–Whitney *U*, chi-square, and Fisher’s exact tests. The multivariate analysis was conducted using forward stepwise (likelihood ratio) multiple logistic regression. The odds ratio (OR) and 95 % confidence interval (95 % CI) were calculated to evaluate the association between risk factors and L5 radiculopathy pathogenesis. Candidate factors (age, sex, and number of fusion levels) were determined by previous studies or were arbitrarily chosen (L5-S1 wedging ≥2° and a foraminal ratio ≤8). *P* < 0.05 was considered as statistically significant.

## Results

### Patient demographics

Patient demographics are presented in Table 1. The mean observation period was 25.2 months. The mean age was 62.2 years in Sym and 64.9 years in Asym, with no significance between groups. Significantly, more intervertebral levels were fused in Sym. Eight of the 125 patients (6.4 %) were categorized to Sym, an average of 13.3 months after the surgery. Two Sym patients underwent revision surgery of L5-S1 TLIF for intractable leg pain from severe postoperative L5-S1 foraminal stenosis 2 years after the surgery with which no conservative treatments were effective.

**Table 1:** Patient demographics

	Sym	Asym	Total
Number of patients (m/f)	8 (6/2)	117 (64/53)	125 (70/55)
Average age (years; mean ± SD)	62.2 ± 12.3	64.9 ± 10.3	66.8 ± 11.3
Average observation period (months)	25.5	24.8	25.2
Average fusion levels (mean ± SD)	1.4 ± 0.96*	1.1 ± 1.02	1.7 ± 0.98
Spondylolisthesis grade (Myerding)			
Grade I	7	109	116
Grade II	1	8	9

*SD* standard deviation, *Sym* symptomatic group, *Asym* asymptomatic group

*P < 0.05 vs. Sym

### Radiographic parameters

**Fig. 2:**
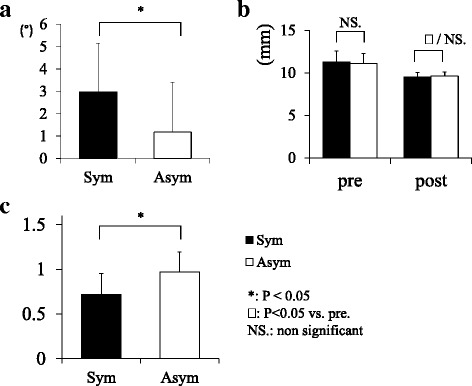
**a** L5-S1 wedging angle was significantly higher in the symptomatic group. **b** Mean disc height decreased without significance in both groups. **c** Preoperative foraminal ratio was significantly lower in the symptomatic group

Preoperative wedging angle was significantly higher in Sym (Sym 2.9° ± 2.2 vs. Asym 1.2° ± 2.2 [mean ± Standard Deviation]; *P* < 0.05; Fig. 2(a)). The mean disc height decreased by 16.4 % (Sym) and 12.3 % (Asym), respectively, but was not significantly different (Fig. 2(b)). Preoperative foraminal ratio was significantly lower in Sym (*P* < 0.05; Fig. 2(c)). No patients showed radiological instability at the final follow-up.

### Risk factors

Multivariate logistic regression analysis revealed that sex (OR 4.61, 95 % CI 2.89–12.5), number of fused levels (5.32, 1.24–58.2), L5-S1 wedging (3.111, 2.9–35.4), and foraminal ratio (4.46, 2.0–10.8) were statistically significant risk factors (Table 2) for L5 radiculopathy.

**Table 2:** Statistical analysis of possible risk factors (multiple logistic regression)

	Odds ratio	95 % CI	*P*
Older age (>65 years)	0.32	0.78–5.61	0.87
Sex (male)^a^	4.61	2.89–12.5	0.005
Number of fused levels (≥2)^a^	5.32	1.24–58.2	0.025
L5-S1 wedging (≥2°)^a^	3.11	2.9–35.4	0.039
Foraminal ratio (≤0.8)^a^	4.46	2.0–10.8	0.031

*CI* confidence interval

^a^Significant risk factor

## Discussion

The significance of the present study was the clinical outcome of L5 radiculopathy due to L5-S1 foraminal stenosis after floating fusion, showing a 6.4 % incidence of L5 radiculopathy after floating fusion. The symptomatic patients showed a significantly larger wedging and smaller foraminal ratio. L5-S1 disc height showed a gradual decrease in both groups with no significance. Multivariate logistic regression detected sex, number of fusion levels, L5-S1 wedging, and foraminal ratio as risk factors for L5 radiculopathy after floating fusion.

Previous studies have mainly focused on ASD per se, while few have examined accompanying neurologic symptoms such as L5 radiculopathy. According to a systematic review of ASD studies, the incidence of ASD and disease associated with lumbar fusion was 34 and 14 %, respectively [12, 13], compared to the present 6.4 % incidence of L5 radiculopathy, one of the major clinical symptoms with ASD after floating fusion. This is a clinically important value to consider in floating fusion.

It has often been reported that L5-S1 disc degeneration increases after fusion surgery compared with decompression only, and longer floating fusion can cause more L5-S1 ASD. One study has reported that 78 % of patients with postoperative ASD had undergone multisegmental fusion surgery [14]. This is consistent with the present study, in which symptomatic patients showed more fused levels compared with asymptomatic patients, suggesting increased loading with a greater number of fused levels. On the other hand, the present study showed a widespread decrease in L5-S1 disc height after the surgery, with no significance between the symptomatic and asymptomatic groups, suggesting a degree of stress loading of the floating fusion.

Furthermore, the occurrence of caudal ASD has been reported to be significantly correlated with pre-existing disc degeneration, potentially increasing the susceptibility of the caudal adjacent disc to ASD [15]. The wedging and narrowed foramen can indicate pre-existing L5-S1 disc degeneration. Our previous study showed that floating fusion surgery caused L5-S1 disc height decrease and consequent foraminal stenosis in one third of 86 patients [16]. The L5-S1 junction should be exclusively considered, because it is more susceptible to significant loading from the trunk, thus increasing stress loading on the L5 pars interarticularis; this is substantiated by another report showing bilateral fractures of the L5 pars after floating fusion in a patient with rheumatoid arthritis [17]. Thus, the present results suggest the significance of lumbosacral wedging as an important risk factor for L5 radiculopathy. The L5-S1 disc height can decrease postoperatively, indicating that the L5-S1 disc with a relatively unchanged height can lead to progressive decrease and degeneration of L5-S1. It is therefore important to consider risk factors for possible postoperative disorders such as L5 radiculopathy as shown in the present study, including increased wedging of the lumbosacral junction and a decreased foraminal ratio.

In the present study, foraminal stenosis was the major cause of postoperative L5 radiculopathy. However, diagnosis of intervertebral foraminal stenosis is inherently difficult. A common method of diagnosis includes the cross-sectional findings of the foramen in the sagittal image with 69 % sensitivity and 54 % specificity [11, 18, 19]. Thus, additional ways of depicting foraminal stenosis, such as diffusion tractography [20] or the ratio suggested in the present study for quantification, should be helpful in the future.

Also, most of the patients were within the spondylolisthesis grade of Myerding I (≤25 %) including the symptomatic case. That indicates the degree of spondylolisthesis did not affect the results.

The present study has some limitations. First, it does not include mobile factors at the dynamic L5-S1 junction, which should be considered in future studies. Second, the present study was retrospective with small sample size. To confirm the results, more patients should be examined prospectively. Third, the risk factors should be more strictly determined with an accompanying cut-off value using a more statistically valid method such as the receiver operating characteristic curve; this should also be considered for future study. Forth, the fusion rate was not exactly evaluated. Future prospective study should include CT scan to evaluate fusion rate. Finally, we did not investigate disc degeneration per se, which can be a potential confounder between the increased disc wedging and decreased foraminal ratio.

## Conclusions

The current retrospective study of 125 patients who underwent lumbar floating fusion surgery showed a 6.4 % incidence of L5 spinal nerve disorder. Higher wedging angle, lower foraminal ratio in the lumbosacral junction, multileveled fusion, and male sex were significant predictive risk factors; spine surgeons should consider an additional lumbosacral fixation for patients with these risk factors, even if the patient has few symptoms from the L5-S1 junction.

## Abbreviations

95%CI: 95 % confidence interval
ASD: adjacent segment disease
Asym: asymptomatic
L: lumbar
MR: magnetic resonance
OR: odds ratio
S: sacrum
Sym: symptomatic
TLIF: transforaminal lumbar interbody fusion
WI: weighted images
